# Advertising Department

**Published:** 1874-04

**Authors:** 


*Advertising Department.*

Attention is called to the several advertisements in the present number. For the good of the profession, we would be pleased to have our advertising columns used as a business directory of reliable houses, etc. As our journal has a large circulation, and we can therefore benefit advertisers and at the same time be of service to the profession, we open our columns to all respectable advertisers who will give us a *quid pro quo* for the privilege. We do not and cannot allow our columns used only upon a legitimate business basis and at fair remunerative rates.

